# Synthesis and Osteoinductive Properties of Nanosized Lithium-Modified Calcium-Organic Frameworks

**DOI:** 10.3390/ma18092091

**Published:** 2025-05-02

**Authors:** Daniel Vargas, Daniel Peña, Emma Whitehead, Warren L. Grayson, Benjamin P. Le Monnier, Michael Tsapatsis, Patricio Romero-Hasler, Rocío Orellana, Miguel Neira, Cristian Covarrubias

**Affiliations:** 1 Laboratory of Nanobiomaterials, Institute for Research in Dental Sciences, Faculty of Dentistry, University of Chile, Santiago 8320000, Chile; daniel.vargas.v@ug.uchile.cl (D.V.); danielpena@odontologia.uchile.cl (D.P.); rorellana@odontologia.uchile.cl (R.O.); mneira@odontologia.uchile.cl (M.N.); 2 Department of Biomedical Engineering, School of Medicine, Institute for Nanobiotechnology, Johns Hopkins University, Baltimore, MD 21201, USA; emma.whitehead@duke.edu (E.W.); wgrayson@jhmi.edu (W.L.G.); 3 Department of Chemical and Biomolecular Engineering, Institute for Nanobiotechnology, Johns Hopkins University, Baltimore, MD 21201, USA; benjamin.lm@jhu.edu (B.P.L.M.); tsapatsis@jhu.edu (M.T.); 4 Department of Food Science and Chemical Technology, Faculty of Chemical and Pharmaceutical Sciences, University of Chile, Santiago 8320000, Chile; patricio.romero@ciq.uchile.cl

**Keywords:** metal-organic frameworks (MOFs), lithium, calcium, osteoinductive nanoparticles, bone regeneration

## Abstract

The development of biomaterials that enhance bone healing and integrate with native bone tissue has gained significant interest. Metal-organic frameworks (MOFs) have emerged as promising candidates due to their unique surface properties and biocompatibility. While various bioactive element-incorporated MOFs have been studied, the osteogenic potential of lithium (Li)-modified MOFs remains largely unexplored. This study presents the synthesis and characterization of a nanosized calcium-based MOF incorporating Li⁺ ions to enhance osteoinductive properties. The MOFs were evaluated in vitro for apatite mineralization, degradation, ion release, protein adsorption, cell adhesion, viability, and osteogenic differentiation using pre-osteoblast cells. The synthesized MOFs promoted apatite formation under simulated physiological conditions, facilitated by their surface nucleation properties, controlled degradation, and sustained Li^+^ and Ca^2+^ ion release. Cytocompatibility assays confirmed excellent pre-osteoblast adhesion and viability. Furthermore, CaMOF nanoparticles stimulated osteogenic differentiation by enhancing alkaline phosphatase (ALP) activity, even in the absence of osteogenic supplements. Among tested MOFs, Li/CaMOF exhibited the highest osteoinductive potential. These findings highlight lithium-modified MOFs as promising biomaterials for bone regeneration. However, further in vivo studies are necessary to assess their long-term stability, bone integration, and clinical applicability.

## 1. Introduction

Bone tissue regeneration and repair remain significant challenges in modern medicine, particularly in conditions such as osteoporosis, fractures, and bone defects resulting from trauma or disease [1]. Globally, over 178 million new bone fractures occur annually, representing a major public health concern [2]. In orthopedics and trauma, these injuries are a leading cause of disability, with treatment costs exceeding USD 400 billion per year [3]. In craniofacial and maxillofacial surgery, bone grafting is required in up to 60% of major reconstructions, highlighting the urgent need for advanced biomaterials to improve bone regeneration and reduce healthcare costs [4]. Traditional approaches, such as autografts and allografts, present several limitations, including restricted availability, risk of immune rejection, and donor site morbidity [5]. Consequently, there is increasing interest in developing novel biomaterials that not only support bone healing but also integrate effectively with native bone tissue. Among these, metal-organic frameworks (MOFs) have gained attention as a promising class of materials due to their high surface area, tunable porosity, structural versatility, and favorable biocompatibility [6].

MOFs are composed of metal ions or clusters coordinated with organic ligands, forming porous crystalline structures with tunable architecture and high surface area. These characteristics allow easy functionalization for specific applications. Recent studies have highlighted their growing relevance in various biomedical fields, including drug delivery, diagnostic imaging, and bone tissue engineering [7,8]. Beyond biomedical uses, MOFs have also shown great promise in the development of ultrasensitive sensor devices, owing to their structural versatility and selective host–guest interactions [9], further expanding their potential for integration in advanced multifunctional platforms. In the biomedical context, several studies have demonstrated the effectiveness of MOFs in promoting bone repair and therapeutic delivery in orthopedic and maxillofacial applications.

For example, MOF coatings on titanium substrates have been shown to enhance bone integration by promoting osteoblast proliferation and bone formation, while also providing antibacterial and anti-inflammatory effects [10,11]. Furthermore, MOFs have been explored as controlled drug delivery systems, enabling the sustained release of agents such as ketoprofen for osteoarthritis, which helps inhibit inflammation and promote bone regeneration [12]. Hybrid composites combining MOFs with polymers or ceramics have also shown enhanced mechanical strength and osteogenic properties [13,14]. In addition, microsized MOF particles formulated with calcium and strontium have been shown to stimulate osteogenic differentiation, while loading them with dimethyloxalylglycine (DMOG) promotes vascular endothelial production [15]. Magnesium/copper MOF coatings on zinc membranes further improve osteogenesis, angiogenesis, and antibacterial properties, aiding bone healing [16].

Despite these advancements, the incorporation of lithium (Li) into MOFs for bone regeneration remains largely unexplored. Lithium is well known for its osteoinductive properties, such as enhancing bone mineral density and accelerating bone formation, particularly through modulation of the Wnt/β-catenin signaling pathway [17,18]. However, its integration into MOF structures has not been yet investigated. Incorporating lithium into MOFs could provide dual benefits: enhancing the osteogenic properties of the material while offering a controlled release system for lithium ions, further promoting bone healing. Additionally, MOF particles with nanometric dimensions could enhance bioactivity by increasing surface area and improving cellular interactions, offering a promising approach to advancing current strategies for bone regeneration.

However, MOFs synthesized with monovalent cations as metal centers often exhibit instability in aqueous media, leading to gradual decomposition and collapse of their structures [19]. A potential strategy to address this instability is to modify the surface of MOFs constructed with divalent metals by introducing lithium ions. This modification could produce a material with osteoinductive properties while maintaining the structural integrity and stability of the MOF framework.

In this study, we investigate the synthesis of nanosized MOF particles with calcium as the metal center, modified with lithium ions to enhance their osteogenic potential, and, for the first time, report their in vitro osteoinductive effects.

## 2. Materials and Methods

### 2.1. Synthesis of Metal Organic Frameworks (MOFs)

**Figure 1:**
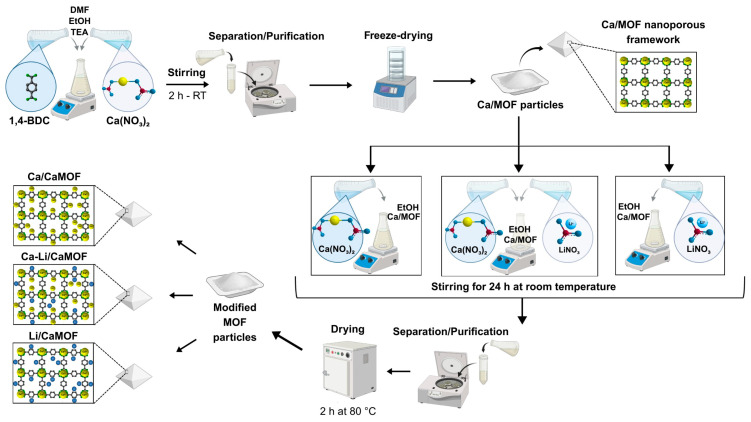
Schematic illustration of the synthesis and post-synthetic modification of CaMOF. CaMOF particles were first obtained by reacting Ca(NO_3_)_2_·4H_2_O with 1,4-BDC in a mixture of DMF, ethanol, and TEA at room temperature, followed by centrifugation and freeze-drying. Post-synthetic modification was carried out by incubating CaMOF with Ca(NO_3_)_2_ and/or LiNO_3_ solutions to obtain Ca/CaMOF, Li/CaMOF, and CaLi/CaMOF.

The synthesis of a calcium-based metal-organic frameworks (CaMOF) was carried out using 1,4-benzenedicarboxylic acid (1,4-BDC, 98% Sigma, Setagaya, Japan) as the ligand and a solvent mixture of dimethylformamide (DMF, 99.8% Sigma, Setagaya, Japan) and triethylamine (TEA, 99.5% Merck, Rahway, NJ, USA) (Figure 1). The solvent mixture was prepared by combining DMF and TEA in a volumetric ratio of 9:1.

After thorough homogenization, the solution was divided into two equal volumes of 63.9 mL each. In the first volume, 7.31 g of calcium nitrate tetrahydrate (Ca(NO_3_)_2_·4H_2_O, Merck) was dissolved to form the metal-containing solution. In the second volume, 2 g of 1,4-BDC was dissolved to prepare the organic ligand solution. After confirming the complete dissolution of both reagents, the metal solution was gradually added to the ligand solution, and the mixture was stirred continuously for 2.5 h at 300 rpm at room temperature.

The resulting mixture was subjected to four centrifugation cycles at 12,000 rpm, followed by two washes with DMF and two washes with 95% ethanol to remove any unreacted components. The final product was frozen at −80 °C for 20 min and then lyophilized for 24 h to yield the CaMOF. The overall reaction yield, calculated based on the initial amount of 1,4-benzenedicarboxylic acid (BDC) as the limiting reagent, was 67.1%.

The CaMOF was surface-modified with lithium ions (Li/CaMOF), calcium ions (Ca/CaMOF), and a mixture of both calcium and lithium ions (CaLi/CaMOF). A 0.1 M ion solution was prepared in 70% ethanol. For the lithium-ion solution, 1.648 g of lithium acetate (CH_3_COOLi, Sigma, Burlington, MA, USA) was dissolved in 250 mL of 70% ethanol. For the calcium ion solution, 5.905 g of calcium chloride (CaCl_2_, Scharlau, Barcelona, Spain) was dissolved in 250 mL of 70% ethanol. The Ca-Li solution was prepared by dissolving 5.905 g of CaCl_2_ and 1.648 g of CH_3_COOLi in 250 mL of 70% ethanol. In the case of the lithium-ion solution, the pH was adjusted to 7.2 with hydrochloric acid to prevent precipitation of hydroxides.

For ion modification, 1 g of MOF was exposed to 100 mL of the respective ion solution in an Erlenmeyer flask under magnetic stirring at room temperature for 24 h. After the impregnation period, the MOF samples were separated by centrifugation at 12,000 rpm and washed four times—twice with 70% ethanol and twice with distilled water. The modified MOFs were then frozen at −80 °C for 20 min and lyophilized to obtain the final products.

### 2.2. Structural Characterization of MOFs

The structural properties of the MOF particles were investigated using X-ray diffraction (XRD) with an SAXSPoint 2.0 SAXS/WAXS system (Anton Paar, Graz, Austria), and scanning electron microscopy (SEM) coupled with X-ray dispersive energy elemental microanalysis (EDX, Aztec EDS, Oxford Instruments, Abingdon, UK), using a JSM-IT300LV microscope (JEOL, Tokyo, Japan) operated at an accelerating voltage of 30.0 kV. The lithium content in the MOFs was quantified via flame emission photometry, using a PFP7 flame photometer (JENWAY, Staffordshire, UK) at the lithium emission wavelength of 671 nm. Physisorption measurements were carried out with an Autosorb iQ analyzer (Anton Paar— Quantachrome, Boynton Beach, FL, USA), employing argon as the adsorbate at a temperature of 87 K. The apparent surface areas (Sg) were determined using the BET equation.

### 2.3. In Vitro Bioactivity Assay

The capacity of the MOFs to promote apatite formation was evaluated in acellular simulated body fluid (SBF), which mimics the ion concentrations of human extracellular fluid. The SBF solution was prepared according to the protocol established by Kokubo et al. [20], using the following standard ion composition (mM): Na^+^ 142.0, K^+^ 5.0, Mg^2+^ 1.5, Ca^2+^ 2.5, Cl^−^ 147.8, HCO_3_^−^ 4.2, HPO_4_^2−^ 1.0, and SO_4_^2−^ 0.5. The solution was buffered at a physiological pH of 7.4 and maintained at 37 °C with tri-(hydroxymethyl) aminomethane (Tris) and hydrochloric acid. Prior to the assay, the MOF powders were compacted into disks using a hydraulic press (CrushIR™, PIKE, Madison, WI, USA) at 9 tons of pressure, resulting in disks with a diameter of 9 mm and a thickness of 2 mm. These MOF disks were individually immersed in 50 mL of SBF in polyethylene containers and incubated at 36.5 °C using a thermostatic water-bath shaker. After incubation period of 14 days, the samples were removed from the SBF, rinsed with distilled water, and dried at 50 °C. The formation of apatite on the MOF surface was assessed using scanning electron microscopy (SEM), EDX elemental analysis, and Fourier-transform infrared (FTIR) spectroscopy with an Agilent Cary 630 ATR-FTIR spectrometer (Santa Clara, CA, USA).

### 2.4. Protein Adsorption Assay

The protein adsorption capacity of the MOFs was determined using bovine serum albumin (BSA) (Thermo Scientific™, Waltham, MA, USA) and fibrinogen from human plasma (Sigma-Aldrich, Burlington, MA, USA) as model proteins. A 10 mL buffered solution (pH 7.4; K_2_HPO_4_/KH_2_PO_4_; 100 mM) containing 200 mg/L of protein was mixed with 100 mg of MOF powder in a glass vial and incubated for 24 h using a thermostatic water-bath shaker. After 24 h of incubation, the samples were centrifuged at 2600 rpm, and the protein concentration in the supernatant was determined using the colorimetric micro bicinchoninic acid (BCA) assay kit (Thermo Scientific™, Waltham, MA, USA). The protein adsorption capacity of the MOFs was calculated using the equation

*Protein adsorption capacity* (%) = (*m_o_* − *m_f_*/*m_m_*) × 100

where *m_o_* is the mass of protein in the initial solution, *m_f_* the mass of protein in the supernatant after 24 h of incubation, and *m_m_* the mass of MOFs.

### 2.5. MOFs Degradation Test

The degradability of the MOFs was assessed by immersing 0.1 g of MOF in 10 mL of Tris buffer pH 7.4 at 37 °C for 28 days. After the incubation period, the MOF powder was separated by centrifugation and dried for 12 h at 70 °C. The material degradation was calculated using the normalized difference between the initial weight *W_o_* and the weight *W_t_* after time t, according to the equation

*Degradation* (%) = ((*W_o_* − *W_t_*)/*W_o_*) × 100

### 2.6. Ion Release Measurements

The release of Li and Ca ions from MOFs was assessed in phosphate-buffered saline (PBS) (pH 7.4) at 37 °C. For this, 0.05 g of MOFs were incubated with 10 mL of PBS for up to 14 days. To determine the Li ion concentration, the MOF particles were first separated by centrifugation, and the ion concentration in the supernatant was measured using flame emission photometry. For calcium, the concentration of Ca ions was directly measured in the MOF/PBS suspension at various time points using a Calcium Combination Ion Selective Electrode (Cole-Parmer EX-27077-02, Vernon Hills, IL, USA).

### 2.7. Cell Culture

The murine calvarial pre-osteoblast cell line MC3T3-E1 (ECACC, 99072810, Salisbury, UK) was utilized to evaluate cell viability, adhesion, and differentiation in the presence of MOFs. MC3T3 cells were grown in Alpha Modified Eagle Medium (α-MEM; GIBCO, Carlsbad, CA, USA) supplemented with 10% fetal bovine serum (FBS; GIBCO, Carlsbad, CA, USA), 100 U/mL penicillin, 10 μg/mL streptomycin, and 0.25 μg/mL amphotericin until the culture reached approximately 80–90% confluence. Once this was achieved, the monolayer of cells was detached from the culture flasks, and the cell density was adjusted to around 50 × 10^3^ cells/mL using α-MEM with 10% FBS. Before exposure to MOF particles, the cells were seeded into 48-well culture plates and incubated overnight at 37 °C in a humidified atmosphere with 5% CO2.

#### 2.7.1. Cell Viability Assays

MOF particles were initially exposed to UV light for 30 min and then suspended in culture medium buffered with 70 mM HEPES at a stock concentration of 1750 µg/mL. To improve particle dispersion, the solution was sonicated for 45 min. The stock solution was then diluted with culture medium to obtain a final concentration of 250 µg/mL.

For the cell viability assay, 50 × 10^3^ cells were plated in a single well of a 48-well plate and treated with the 250 µg/mL MOF concentration for 3, 7, and 14 days. Cell viability was measured using the CellTiter 96^®^ Aqueous One Solution Cell Proliferation Assay (Promega, Madison, WI, USA), which quantifies the reduction of [3-(4,5-dimethylthiazol-2-yl)-5-(3-carboxymethoxyphenyl)-2-(4-sulfophenyl)-2H-tetrazolium] (MTS) to formazan by viable cell mitochondria. After the incubation period, MTS reagent was added to each well, and the plate was incubated for 1 h at 37 °C. Absorbance was measured at 490 nm using an ELISA microplate reader (Tecan Infinite F-50, Zürich, Switzerland). A control group of MC3T3 cells without MOF particles was included for comparison.

#### 2.7.2. Cell Adhesion Assays

Cell adhesion to the MOF particles was evaluated after 48 h of incubation. Cells were fixed with 4% paraformaldehyde for 20 min at room temperature. To visualize the cytoskeleton and nuclei, the cells were stained with rhodamine-phalloidin and 4,6-diamidino-2-phenylindole (DAPI) for 1 h in the dark. Imaging was conducted using a Zeiss Z2 Axio Observer.

#### 2.7.3. Cell Differentiation Assays

The ability of MOF particles to promote cell differentiation into an osteogenic lineage was assessed by measuring alkaline phosphatase (ALP) enzymatic activity after 7 and 14 days of incubation without osteogenic supplements. Cells cultured in osteogenic medium, which consisted of αMEM medium, 10% FBS, 50 μg/mL ascorbic acid, 10 mM sodium 2-glycerophosphate, and 1 μM dexamethasone, served as the positive control.

ALP activity was quantified using a colorimetric dephosphorylation assay with the p-nitrophenyl phosphate (pNPP, Merck, Rahway, NJ, USA) reagent at 405 nm, following the manufacturer’s instructions. In addition, ALP activity was evaluated through staining with nitroblue tetrazolium (NBT, Roche, Basel, Switzerland) and 5-bromo-4-chloro-3-indolyl phosphate p-toluidine (BCIP, Roche, Basel, Switzerland). Cells were first rinsed with phosphate-buffered saline (PBS), fixed with 4% paraformaldehyde in ethanol for 30 s, and then stained with NBT/BCIP for 12 h at 4 °C. Stained areas were observed under a microscope, and the images were analyzed and quantified using ImageJ^®^ software (version 1.53t, National Institutes of Health, Bethesda, MD, USA).

### 2.8. Statistical Analysis

Data from at least three independent experiments were analyzed and are presented as mean ± standard deviation. Differences between groups were assessed using one-way ANOVA, followed by Tukey’s post hoc test. A *p*-value of less than 0.05 was considered statistically significant. All statistical analyses were performed using Origin 2024 software.

## 3. Results and Discussion

### 3.1. Synthesis and Characterization of MOFs

**Figure 2:**
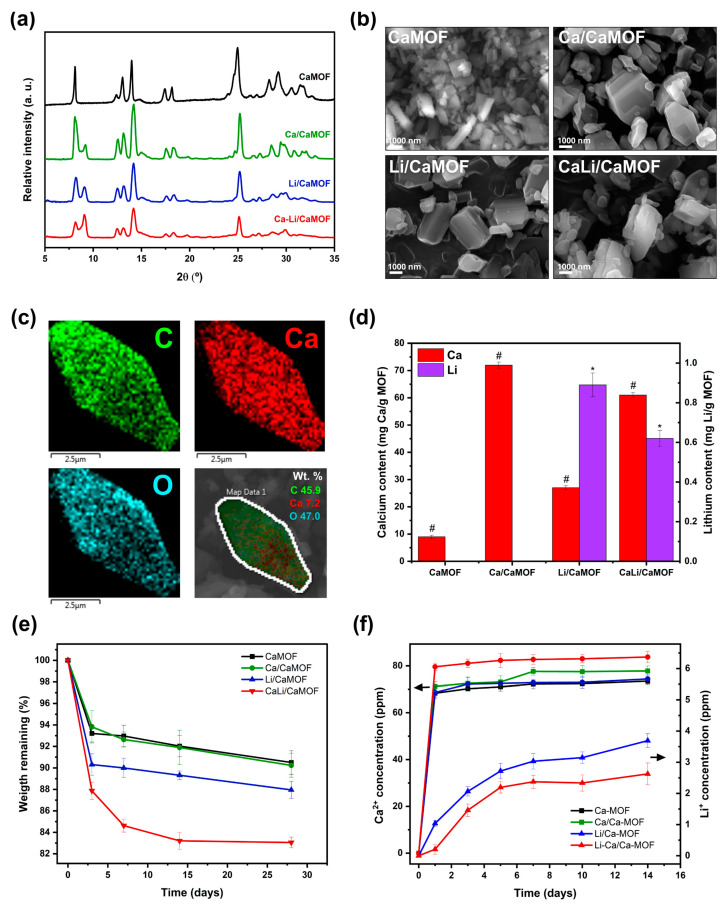
Characterization of the synthesized MOFs: (**a**) SEM images and (**b**) XRD patterns of the MOF particles, (**c**) EDX elemental mapping of a Ca/CaMOF particle, (**d**) calcium and lithium contents in the MOFs, (**e**) weight loss curves, and (**f**) calcium and lithium ion release profiles of the MOFs in PBS at pH 7.4 and 37 °C. Values are expressed as mean ± SD from three independent experiments. # Indicates statistically significant differences in calcium content, while * denotes statistically significant differences in lithium content (*p* < 0.05).

Figure 2a shows the XRD patterns of the synthesized calcium-based MOFs. The pristine CaMOF displays sharp, well-defined peaks, indicative of a highly crystalline material. These reflections are consistent with those reported for [Ca(BDC)(DMF)(H_2_O)] frameworks synthesized under solvothermal conditions [21,22], which crystallize as three-dimensional networks composed of CaO_8_ polyhedra connected by μ_4_-coordinated terephthalate (BDC) linkers. Despite being synthesized at room temperature, the similarity in diffraction profiles suggests a comparable underlying topology, likely involving zigzag one-dimensional Ca–O chains bridged by BDC units. Following ion exchange with extraframework Li^+^ and/or Ca^2+^, the main diffraction peaks remain largely unchanged, indicating that the long-range crystallographic order of the parent framework is preserved. However, a new reflection appears at ~9° 2θ, absent in the pristine material, suggesting the formation of additional crystallographic planes—potentially due to local rearrangements or partial reordering within the pore channels [22]. Moreover, a reduction in peak intensity is observed in the 28–32° 2θ range for the ion-modified samples. This attenuation likely reflects subtle lattice distortions or internal strain, possibly arising from differences in the ionic radii and coordination geometries of the incorporated cations [23]. Similar framework perturbations have been previously described in Ca–BDC systems, where thermal or hydration-induced changes can alter coordination modes, induce BDC linker rotation, and promote partial reorganization of the framework without compromising its overall crystallinity [21,22].

The synthesized Ca-MOF crystals (Figure 2b) exhibit cubic to truncated octahedral morphologies with average dimensions of ~930 nm in length and ~300 nm in thickness. These nanometric sizes are markedly smaller than those typically reported for calcium-based MOFs synthesized under solvothermal conditions. For example, Jamil et al. [24] described cubic Ca-MOF crystals measuring 5–7 μm when prepared at 110 °C using CaCO_3_ and BDC in DMF/water mixtures. In contrast, our room-temperature synthesis yields submicron particles with well-defined faceting, likely due to kinetic control over crystal growth and the absence of prolonged thermal input. The use of ethanol and DMF as co-solvents may also influence morphology by modulating nucleation and coordination dynamics. The formation of truncated octahedral shapes—uncommon in Ca-MOFs—may result from preferential growth inhibition along specific crystallographic planes, potentially stabilized by solvent–surface interactions during early nucleation. Comparable morphological variations have been reported for other Ca-MOFs. Wang et al. [25] observed a shift from rod-like to needle-like structures depending on acetic acid concentration during synthesis, while Kaur et al. [26] reported uniform nanoplate morphologies (~200 nm) in a bio-CaMOF assembled from aspartic acid. These controlled nanometric geometries, achieved under mild conditions, may enhance the osteoinductive performance of Ca-MOF particles by increasing their surface area and facilitating cellular interactions relevant to bone tissue regeneration. EDX elemental mapping of CaMOF (Figure 2c) confirms the exclusive presence of C, O, and Ca within the structure, with no detectable N atoms, indicating the absence of residual DMF/TEA solvents. The BET specific surface area, measured using argon as the adsorbate to ensure accurate assessment of nanoporosity, yielded values of 53.1, 47.2, 54.6, and 97.1 m^2^/g for CaMOF, Li/CaMOF, Ca/CaMOF, and CaLi/CaMOF, respectively. Despite the removal of solvent molecules, the surface areas remain relatively low, likely due to the triclinic crystal structure of Ca-BDC MOF. This architecture is characterized by narrow pore channels [27], which limit pore accessibility and restrict the diffusion of even small adsorbate molecules such as argon.

Following impregnation with Li^+^ and Ca^2+^, a notable increase in the concentration of these elements is observed (Figure 2d), indicating their successful incorporation onto the CaMOF surface. MOF particles also exhibited partial degradation under physiological pH, with weight loss ranging from 10% to 17% (Figure 2e). Notably, lithium-modified MOFs showed increased degradability, likely due to lithium ions weakening coordination bonds and disrupting the MOF framework by competing with calcium for coordination sites [28]. Furthermore, the MOFs demonstrated the capacity to release Ca^2+^ ions (74–84 ppm) and Li^+^ ions (2.6–3.7 ppm) from lithium-impregnated samples (Figure 2f), particularly after 24 h of immersion at physiological pH.

### 3.2. In Vitro Apatite—Forming Capacity

**Figure 3:**
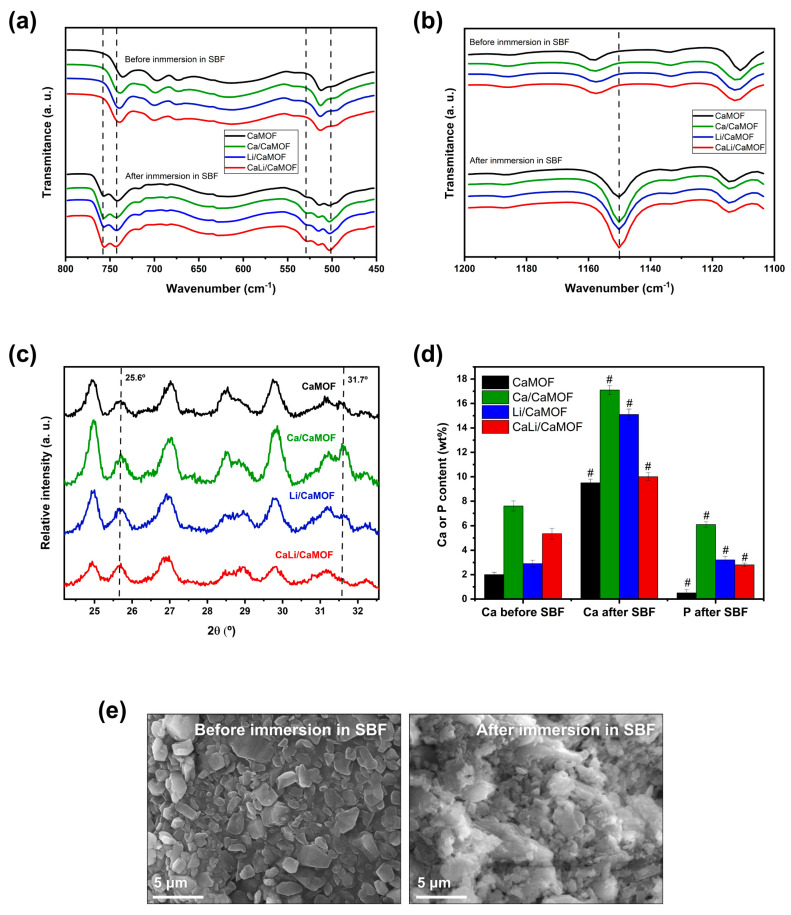
Bioactivity of MOFs in simulated body fluid (SBF) at 37 °C for 14 days. (**a**,**b**) ATR-FTIR spectra showing vibrational bands associated with apatite formation. (**c**) XRD patterns of MOF samples highlighting peaks corresponding to the apatite structure. (**d**) EDX compositional analysis of MOF surfaces showing calcium and phosphorus content before and after SBF immersion. (**e**) Representative SEM images of Li/CaMOF before and after SBF immersion, illustrating surface morphological changes. Values are expressed as mean ± SD from three independent experiments. # Indicates statistically significant differences in calcium and phosphorous content after immersion in SBF (*p* < 0.05).

The ability of the MOFs to promote bone-like apatite mineralization was evaluated in simulated body fluid (SBF) over 14 days. FTIR-ATR spectroscopy (Figure 3a,b), recognized for its sensitivity to bone-like apatite formation [29], revealed the emergence of a doublet at 528 and 502 cm^−1^ after immersion in SBF. These bands are attributed to the P–O bending vibrations of phosphate groups (PO_4_^3−^) characteristic of apatite phases. Although the typical P–O bending doublet is commonly observed around 565 and 605 cm^−1^, downshifts in frequency are often associated with poor crystallinity, cationic substitution, or the presence of amorphous or non-apatitic calcium phosphate phases, as well as hydration-related lattice distortions [30]. Additionally, a band at ~1150 cm^−1^ becomes more intense upon SBF immersion. This band corresponds to symmetric P–O stretching vibrations and further supports the formation of phosphate-containing mineral phases. Beyond the typical phosphate bands associated with apatite, spectral analysis revealed notable features in the 650–725 cm^−1^ and 1110–1120 cm^−1^ regions. In the 650–725 cm^−1^ range, a weak band centered around 710–720 cm^−1^ was observed in the pristine Ca-BDC MOF, which is consistent with out-of-plane C–H bending modes of the aromatic ring in the BDC ligand. According to Mazaj et al. [21], this band can be modulated by hydration or framework relaxation upon guest exchange. A subtle reduction in this band’s intensity post-immersion may indicate partial masking by mineral deposition or local ligand reorientation at the MOF surface. In the 1110–1120 cm^−1^ region, the appearance of a narrow shoulder prior to SBF exposure can be assigned to asymmetric C–O stretching modes of coordinated carboxylates. This assignment aligns with previous FTIR studies of Ca-BDC MOFs [21,31]. Upon immersion, this feature overlaps with the more intense phosphate band at 1150 cm^−1^, suggesting surface-level interactions between phosphate species and the MOF matrix. Taken together, the observed spectral changes are consistent with the early formation of phosphate-rich phases at the MOF surface, indicative of incipient apatite nucleation triggered by exposure to simulated body fluid.

XRD analysis of the SBF-conditioned MOF surfaces (Figure 3c) revealed the appearance of two characteristic reflections of apatite at 25.6° and 31.7° (2θ), confirming the formation of crystalline apatite on the material surfaces. This mineralization process was further supported by an increase in calcium and phosphorus concentrations, as measured by EDX (Figure 3d). Moreover, SEM imaging revealed evident morphological alterations on the MOF surfaces following 14 days of immersion in SBF (Figure 3e). The originally smooth and well-faceted particles became partially covered by fine granular deposits forming discontinuous layers and globular aggregates. These surface features are indicative of calcium phosphate precipitation and are characteristic of early-stage apatite nucleation on bioactive materials [32]. Although SBF-induced apatite typically exhibits needle-like or spheroidal morphologies at the onset of nucleation, these can gradually evolve into denser and more extensive mineral layers over time [33,34]. In the present study, the observed mineral phase appears as localized surface deposits rather than a fully continuous coating, suggesting that surface mineralization remains in progress and occurs in a spatially heterogeneous manner.

The ability of MOF particles to directly induce apatite formation in vitro had not been reported previously, except by Moris et al. [35], who observed that gelatin scaffolds loaded with a zirconium-based metal-organic framework (MOF 801) produced apatite mineral deposits after 28 days of immersion in SBF. Apatite crystallization in SBF fundamentally depends on factors that govern the thermodynamically stable formation of apatite crystals, including the presence of nucleating particles, variations in local supersaturation, surfaces with chemical functional groups, among others [36]. In the current study, CaMOF demonstrated the ability to form apatite, which appeared to be enhanced by the presence of Ca^2+^ and Li^c^ on its surface, as indicated by the increased calcium concentration and the appearance of phosphorus after SBF immersion. Thus, the SBF bioactivity of the MOFs can be attributed to a combination of factors, including the nucleation capacity of the nanosized particles, the exposure of carboxylic groups and the release of Ca^2+^ as the material degrades, as well as their surface area and porosity. Li^+^ ions can also play an indirect role in influencing local saturation, altering surface charge and hydrophilicity, and enhancing the structural stability of the initial apatite nuclei [37,38].

### 3.3. Cytocompatibility

**Figure 4:**
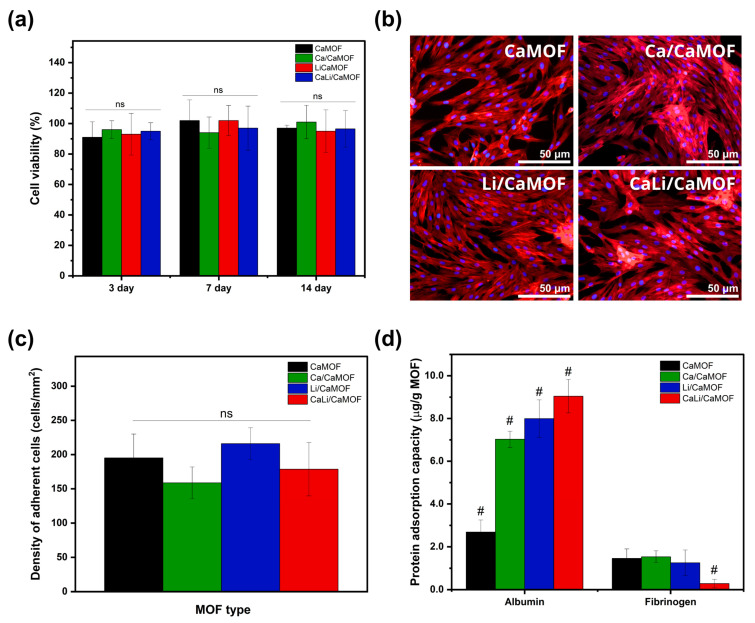
Cytocompatibility assays. (**a**) Viability of MC3T3 cells incubated with MOF particles (250 µg/mL) for 3, 7, and 14 days, assessed using the MTS assay. (**b**) Cell adhesion of MC3T3 cells on MOF particles, visualized by epifluorescence microscopy, with Phalloidin-labeled F-actin (red) and DAPI-stained nuclei (blue). (**c**) Quantification of adherent cells on each MOF. (**d**) Adsorption capacity of fibrinogen and albumin on MOFs. Graphs represent the mean ± SD of three independent experiments. # Indicates significant differences between groups, while “ns” denotes no significant differences (*p* > 0.05).

The cytocompatibility of the different MOF samples was evaluated using pre-osteoblast MC3T3 cells over an incubation period of up to 14 days (Figure 4a). MTS cell viability, expressed as a percentage relative to the control group (cells cultured without particles), remained at 100% for all MOF types. Cells also displayed excellent adhesion to the MOF particles (Figure 4b). Fluorescent staining with rhodamine-phalloidin and DAPI revealed well-organized actin filaments maintaining their integrity, while the nuclei remained intact, showing no signs of fragmentation or condensation. The analysis of cell adhesion density across MOF particles (Figure 4c) showed no significant differences, suggesting that their structure and composition have little impact on cell adhesion. The ability of MOFs to adsorb extracellular proteins was also evaluated (Figure 4d). Albumin, a globular protein with minimal involvement in cell adhesion, exhibited increased adsorption in the presence of calcium and lithium cations on the MOF surface. In contrast, fibrinogen, a protein with an elongated rod-like morphology, showed similar adsorption across all MOF surfaces, with a slight decrease observed on CaLi/CaMOF. These adsorption profiles reflect surface–protein interactions that are often influenced by material hydrophilicity, and may contribute to the observed cell adhesion patterns. The similar fibrinogen adsorption capacity of the MOFs aligns with the comparable cell adhesion observed in these materials. Previously reported studies confirm that MOFs exhibit good cytocompatibility with bone-related cells [10,11,12,15]. In this study, we further demonstrate that nanodimensional MOF particles composed of calcium and lithium support preosteoblastic cell adhesion and proliferation.

### 3.4. Cell Differentiation

**Figure 5:**
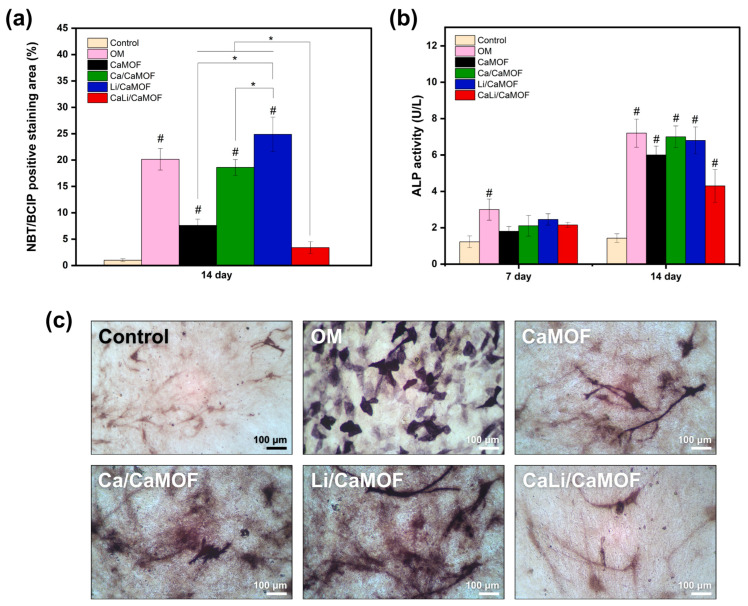
Osteogenic cell differentiation assays. (**a**) Alkaline phosphatase (ALP) activity in MC3T3 cells, measured using the pNPP method after 7 and 14 days of incubation without material (Control), with osteogenic medium (O.M.), or with MOF particles. (**b**) Quantification of ALP-positive staining using the NBT/BCIP method. Values are expressed as mean ± SD from three independent experiments. # Indicates significant differences compared to the Control group (*p* < 0.05). * Indicates statistically significant differences between experimental groups (*p* < 0.05). (**c**) Representative ALP staining images obtained with the NBT/BCIP method.

The osteogenic differentiation potential of MOF particles was assessed by analyzing alkaline phosphatase (ALP) activity in MC3T3 preosteoblast cells. The MC3T3-E1 cell line is widely used as an in vitro model for bone formation due to its ability to differentiate into mature osteoblasts and produce a mineralized bone matrix [39]. In these experiments, MC3T3 preosteoblasts were cultured with MOFs in the absence of osteogenic supplements, ensuring that differentiation was driven exclusively by the zeolitic nanoparticles. After 7 days of incubation, ALP activity, assessed using the pNPP method (Figure 5a), showed a significant increase only in cells treated with the osteogenic medium (O.M.). However, by day 14, cells cultured with MOF particles exhibited significantly higher ALP activity compared to the control group, although no significant differences were observed among the MOF-treated groups. ALP activity was further validated using the NBT/BCIP method (Figure 5b,c), confirming that only CaMOF, Ca/CaMOF, and Li/CaMOF significantly enhanced ALP activity compared to the control. Notably, Li/CaMOF exhibited the highest ALP activity, surpassing that of Ca/CaMOF. Unlike the pNPP assay, BCIP/NBT forms an insoluble precipitate at the enzyme activity site, allowing direct visualization and enhancing sensitivity, particularly for detecting low ALP levels [40]. This localized reaction prevents signal diffusion, a common limitation of soluble assays like pNPP. Since ALP is expressed during osteoblast-mediated extracellular matrix deposition, it serves as a reliable marker of osteogenic differentiation induced by MOF particles [41].

The ability of CaMOF and Ca/CaMOF to promote osteogenic differentiation suggests that nanotopographical features, such as porosity and surface characteristics, along with calcium release, play a crucial role in this effect. The osteoinductive properties of nanoporous materials have been extensively documented [42,43]. Moreover, calcium ions released from Ca-based MOFs can activate specific intracellular signaling pathways involved in osteogenic differentiation, such as SMAD1 phosphorylation—a critical step in triggering bone morphogenetic protein (BMP)-related gene expression [44]. On the other hand, CaMOF modified with surface-adsorbed Li^+^ ions (Li/CaMOF) exhibited an even greater osteoinductive effect, which can be attributed to the ability of released Li^+^ ions to stimulate osteogenic differentiation by inhibiting GSK-3β, stabilizing β-catenin, and activating the Wnt/β-catenin signaling pathway [17,45]. Surprisingly, CaMOF modified with both Ca^2+^ and Li^+^ (CaLi/CaMOF) showed a lower osteoinductive effect, potentially due to its higher degradability (Figure 2e). The increased degradation may compromise its porous architecture, thereby reducing its osteoinductive nanotopographic properties.

While lithium doping in bioceramics has been proposed as a promising strategy for developing more osteoinductive biomaterials [46], the results obtained with Li/CaMOF particles provide the first evidence of MOFs modified with lithium for bone tissue repair applications. The surface properties of MOFs allow the controlled release of lithium ions, maintaining therapeutic levels over extended periods. Additionally, lithium suppresses osteoclastogenesis, which could further enhance bone regeneration [47].

Lithium-modified MOFs exhibit promising osteoinductive properties, making them attractive candidates for bone-related biomedical applications. However, given the limitations of in vitro assays in replicating the physiological complexity of bone tissue, further in vivo studies are essential. Evaluating these MOFs in a bone defect model will provide critical insights into their integration, biodegradability, and long-term regenerative potential, bridging the gap between in vitro findings and clinical applicability.

## 4. Conclusions

This study demonstrates the successful synthesis and bioactivity of nanosized lithium-modified calcium-based MOFs. These materials showed excellent cytocompatibility, promoted osteogenic differentiation via enhanced alkaline phosphatase (ALP) activity, and facilitated bone-like apatite formation in SBF. Among the tested materials, Li/CaMOF exhibited the highest osteoinductive potential. These findings highlight lithium-modified MOFs as promising candidates for bone regeneration. Further in vivo studies are needed to evaluate their long-term stability, bone integration, and clinical applicability. In addition, their application as osteoinductive agents in composite scaffolds, injectable systems, biofunctional implant coatings, and 3D-printed constructs should be explored, leveraging their nanoscale dimensions, surface functionality, and ion-release properties for advanced bone tissue engineering strategies.

## Data Availability

No new data were created or analyzed in this study. Data sharing is not applicable to this article.
